# The good, the bad, and the ugly: Evolutionary and pathological aspects of gene dosage alterations

**DOI:** 10.1371/journal.pgen.1009906

**Published:** 2021-12-09

**Authors:** M. Felicia Basilicata, Claudia Isabelle Keller Valsecchi

**Affiliations:** Institute of Molecular Biology (IMB), Mainz, Germany; Fred Hutchinson Cancer Research Center, UNITED STATES

## Abstract

Diploid organisms contain a maternal and a paternal genome complement that is thought to provide robustness and allow developmental progression despite genetic perturbations that occur in heterozygosity. However, changes affecting gene dosage from the chromosome down to the individual gene level possess a significant pathological potential and can lead to developmental disorders (DDs). This indicates that expression from a balanced gene complement is highly relevant for proper cellular and organismal function in eukaryotes. Paradoxically, gene and whole chromosome duplications are a principal driver of evolution, while heteromorphic sex chromosomes (XY and ZW) are naturally occurring aneuploidies important for sex determination. Here, we provide an overview of the biology of gene dosage at the crossroads between evolutionary benefit and pathogenicity during disease. We describe the buffering mechanisms and cellular responses to alterations, which could provide a common ground for the understanding of DDs caused by copy number alterations.

## Introduction

In sexually reproducing eukaryotes, genes are typically present in 2-fold, a maternal and a paternal complement. A characteristic feature of this balanced karyotype is that the chromosomes are present in proportional amounts with respect to each other. The relative copy number of a gene in the genome is referred to as gene dosage. Changes in gene dosage proportionally reflect on RNA and protein products and thereby carry the potential to affect cellular processes in which they play a role, both in a detrimental or beneficial fashion. Aneuploidy refers to an imbalanced chromosome complement and is only rarely tolerated during embryonic development. It is a principal factor of implantation failure and perinatal lethality in humans with the notable exception of trisomy of chromosome 21, where affected individuals have a life expectancy of about 60 years [1]. Gene dosage changes can also occur naturally, for example, in the form of polyploidy, which is a widespread phenomenon in plants and also found in fishes, amphibians, reptiles, and insects [2]. Another example are heteromorphic sex chromosomes, where degeneration of the Y/W can result in only a single functional allele being present in the heterogametic sex. In some organisms, the resulting reduction in expression of sex-linked genes is reequilibrated by a mechanism termed “dosage compensation” (DC) [3].

Albeit these chromosome-wide examples are probably the most well-known ones, gene dosage alterations can occur at any level, from the mega-base scale down to the individual gene. Whole chromosomes can be gained or lost, parts of chromosomes containing one or many genes can be amplified or deleted, and, furthermore, one of the 2 gene copies can be inactivated by heterozygous mutations. Recent advances in diagnostics by next generation sequencing (NGS) brought forward a growing number of rare developmental disorders (DDs) caused by de novo variants in single genes [4]. Large-scale sequencing efforts of the healthy population, in turn, revealed that at least 20% of the human coding genome is highly intolerant to heterozygous mutation [5,6]. This emphasizes that gene dosage imbalance has a much bigger impact for human health than anticipated.

**Fig 1:**
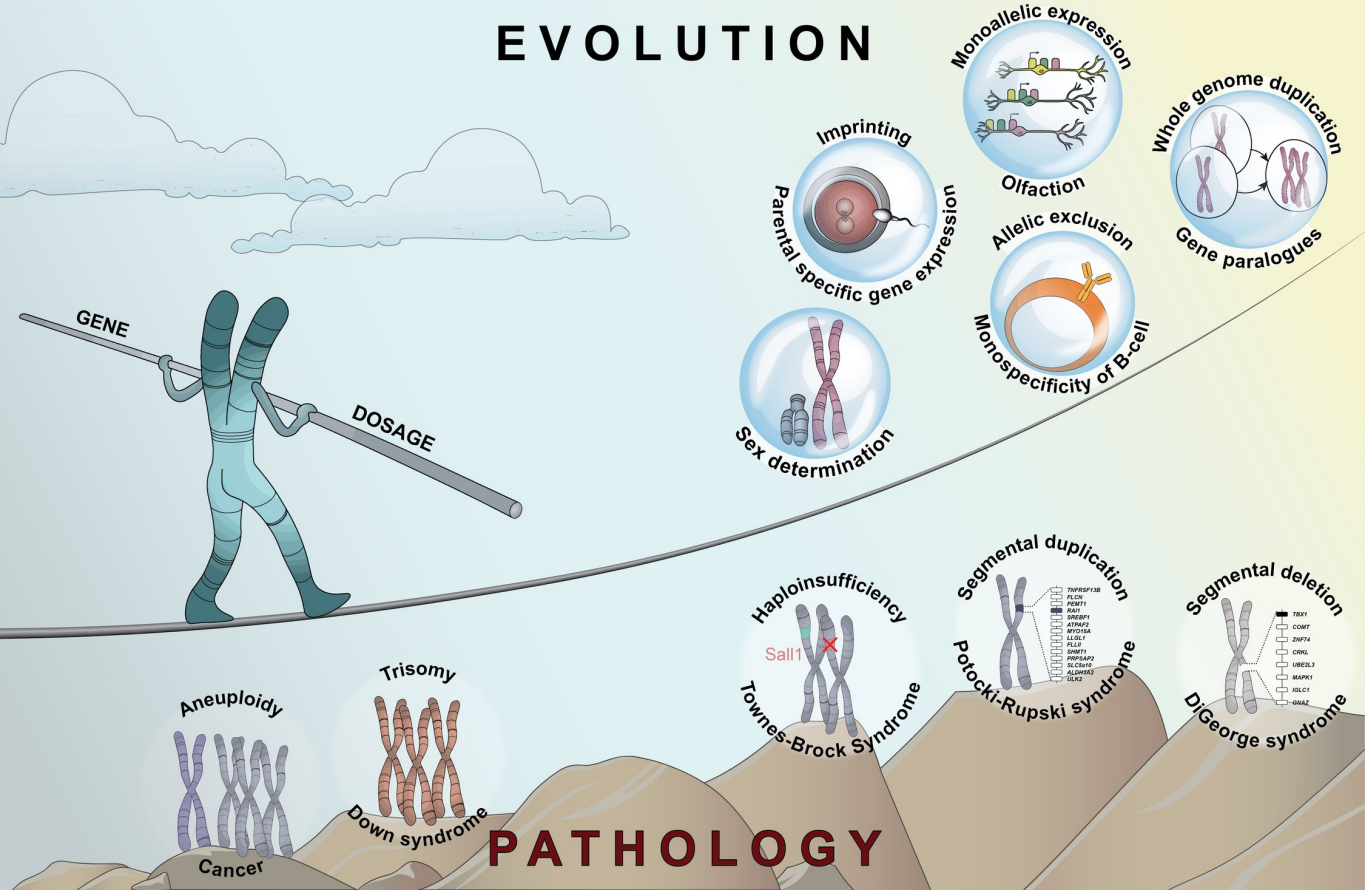
Overview of chromosome-wide to gene-by-gene dosage alterations. Artistic representation of the beneficial and the disadvantageous aspects of gene dosage alterations. Examples of naturally occurring and disease-causing phenomena are shown in the illustration. Such gene dosage alterations can occur from any level, from the chromosome-wide, i.e., mega-base scale, down to the individual gene level, i.e., a single-nucleotide change. Dosage can be modulated by genetic and epigenetic mechanisms.

Here, we provide an overview of the evolutionary and pathological aspects of gene dosage alterations and their impact on the outcome of development (Fig 1). Building on the knowledge about sex chromosome regulation, we discuss other forms of expression imbalances, the associated perturbations of molecular and cellular networks as well as response mechanisms. For an in-depth discussion of the diverse facets of gene dosage related to cancer, we refer to recent reviews [7,8].

### Chromosome-wide dosage alterations: Aneuploidies and sex chromosomes

The importance of maintaining appropriate gene dosage balance was noted by Blakeslee and colleagues in their experiments performed in the 1920s with the plant *Datura stramonium*. He found that in a balanced genome, addition of a single chromosome leads to detrimental effects. Whole genome duplication by hybridization instead did not cause phenotypic changes [9]. Similar findings were later obtained in, e.g., other plants, fruit flies, yeast [10–12], and humans. A systematic analysis of segmental aneuploidies in fruit flies revealed that viability and fertility are compromised when the heterozygous deletion spans approximately 1% of the genome. Heterozygosity from 3% up to entire chromosomes results in embryonic lethality [11,13]. Tissue-specific aneuploidies promote a variety of phenotypes in the fruit fly, including a decreased life span, defects of the nervous system, or perturbed stem cell proliferation [14–16]. In mice, highly aneuploid embryonic stem cells fail to contribute to all tissues of the adult chimera including the germline [17]. Aneuploidy in plants can lead to leaf knotting, aberrant branching, growth defects, and general developmental delay [18,19]. Aneuploid budding yeast exhibits proliferative defects, alterations in cell division and cell cycle kinetics, plasma membrane stress, defects in responding to environmental perturbations [20,21], and are accompanied by the induction of a general stress response, proteotoxicity, and genome instability [8] (Box 1). Thus, aneuploidies and the associated imbalances can negatively affect cellular and organismal fitness and are often not tolerated during development.

**Box 1. Cellular and molecular responses upon aneuploidy**

The gene expression changes after chromosomes become aneuploid can trigger alterations in cellular function. Traditionally, it was assumed that such responses are gene specific, and, hence, the cellular consequences upon aneuploidy differ depending on which genomic region exhibits a copy number change. However, it is important to note that aneuploidy also triggers global physiological and molecular effects, regardless of which specific chromosome and encoded genes are involved [10,11,21]. It has been suggested that by affecting many genes simultaneously, aneuploidy is an extra burden for cellular machineries such as the proteostasis network, which is responsible for maintaining the proper balance of protein synthesis, folding, transport, and degradation [57]. Aneuploidy can disrupt cellular metabolism due to a higher metabolic demand linked to increased nucleotide, protein, and lipid synthesis. Besides this “extra work” aspect, overdosage of balanced protein complexes [114] but also unpaired multisubunit complexes can cause aggregation of proteins, which provides an alternative route to protein degradation to respond to gene dosage imbalance [115] (also see Fig 3).

**Fig 2:**
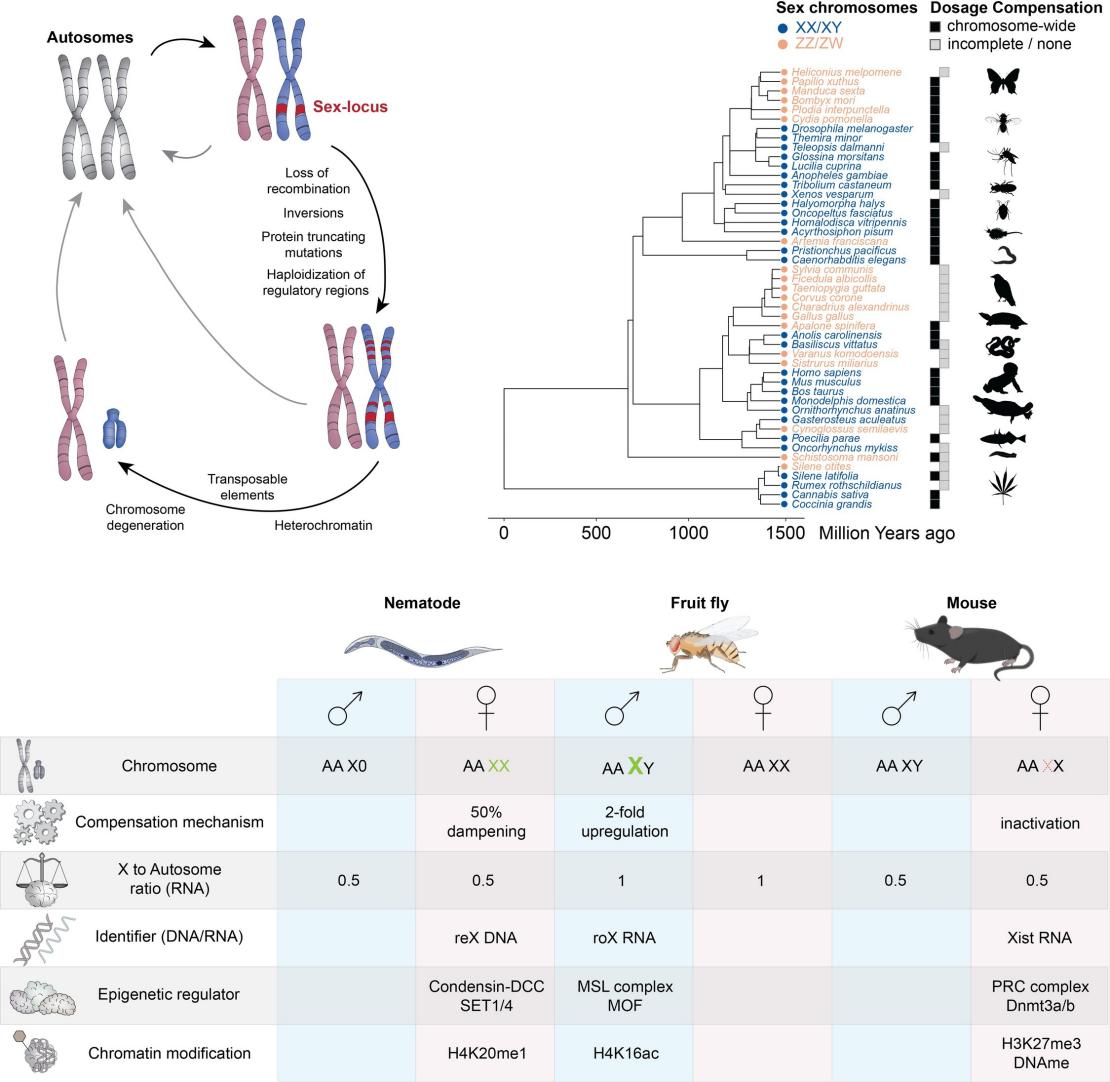
Sex chromosomes and DC. (Top left) Sex chromosomes are highly dynamic and show recurrent turnovers illustrated by gray arrows. They typically evolve from a pair of regular autosomes after acquisition of a sex-determining locus. Recombination starts to be repressed and the future Y (in the case of a male heterogametic species) or W (in the case of a female heterogametic species) acquire more and more protein truncating mutations. This process results in gradual functional heterozygosity of the X or Z chromosome (pink). In some organisms, the sex chromosomes then become fully degenerated and are sometimes even entirely lost, but there are also many species, where sex chromosomes do not decay [37]. Despite degeneration, some genes can be retained [41] or even become expanded on the degenerating Y/W [108]. (Top right) Evolutionary tree showing multiple species across the animal and plant kingdom, where DC has been studied. XY and ZW sex chromosome systems are colored in blue and orange, respectively, and the presence of chromosome-wide versus gene by gene/absence of DC are illustrated with black and gray boxes. Pictograms (images: phylopic.org) are only shown for illustrative purposes and do not depict the actual species in the tree; also see references and comments in S1 File. (Bottom) Comparison of the 3 known molecular mechanisms achieving DC by up-regulation of the X in males (*Drosophila*), inactivation of the X in females (mammals) or dampening of the 2 X by half in hermaphrodites (nematodes) are compared in the table. In *Drosophila*, both X-to-autosome as well as male-to-female DC is reached. Mammalian females undergo X chromosome inactivation, where besides selection to correct for dosage imbalance, sexual antagonism has been proposed as an alternative mechanism shaping X inactivation during evolution [109]. Whether the remaining, active X and the single male X are globally up-regulated by 2-fold remains ambiguous to date. While transcriptional mechanisms have been broadly investigated [110–112], a recent study comparing different vertebrates suggests that this second level of compensation according to Ohno’s hypothesis is achieved via translational regulation [113]. DC, dosage compensation.

However, there are interesting physiological exceptions: Aneuploidy appears to be part of a developmental program of the mammalian liver and brain [2,22]. Another naturally tolerated aneuploidy are the heteromorphic sex chromosomes (XX/XY and ZZ/ZW), which are present in many sexually reproducing organisms and evolve from regular autosomes [23] (Fig 2, top left). According to Ohno, correction of the expression imbalances due to X chromosome monosomy versus autosomes requires an up-regulation by 2-fold. He also postulated that in order to balance differences between sexes and to achieve complete DC, females would require X chromosome inactivation [24]. With regard to the current usage of the DC term, it is helpful to consider these 2 sides of the coin of Ohno’s hypothesis: X-to-autosome balance versus X-to-X balance between the sexes (Fig 2, bottom; also see [25]).

Only 3 molecular pathways to achieve DC have been characterized in detail: the ones operating in mammals, fruit flies, and nematodes (for reviews, see [3,26,27]). Of note, the macromolecular complexes and associated transcription level changes in these species are entirely distinct (Fig 2, bottom). Phenotypically, the genetic loss of the factors promoting DC result in sex-specific lethality at larval stages in *Caenorhabditis elegans* and *Drosophila melanogaster* [28,29]. In mice, depending on the genetic allele investigated, lethality manifests either during embryonic stages or at weaning [30,31]. Whether these phenotypes are a consequence of imbalance of selected dosage-sensitive genes or reflect a particular sensitivity of these species to an aneuploid chromosome remains only poorly characterized to date, especially with regard to the temporal and tissue-specific requirements of DC.

Besides these model organisms, the presence or absence of DC has been documented in many other species encompassing not only the animal, but also the plant kingdom (Fig 2, top right; S1 File) [32]. Across taxa, there can be a remarkable complexity and diversity of the associated mechanisms. For example, the two dipterans *Anopheles* and *Drosophila* achieve X chromosome up-regulation in males by entirely different mechanisms, although the same ancestral autosome evolved to a sex chromosome [33], and the same ancestral segment turning into a sex chromosome is compensated in XY anolis lizards but not the ZW softshell turtle [34]. DC can be regulated depending on developmental stage, tissue, and age: In the parasite *Schistoma mansoni*, the Z chromosome is fully compensated in only the free, cercaria stage, but not in its parasitic, intravertebrate stage [35] (also see an interesting example of tissue-specific DC in turtles [36]). Many organisms do not display chromosome-wide expression equalization of sex chromosomes [37]. In chicken, which possesses a ZZ/ZW sex determination system, only few dosage-sensitive genes are subjected to DC [38,39], and the snake Z chromosome lacks chromosome-wide DC, too [40]. DC may not be necessary, if dosage balance is maintained by keeping 2 functional copies in both sexes, for example, via the “survival” of especially dosage-sensitive genes on the degenerating W or Y [41,42]. An emerging picture is that chromosome-wide DC occurs much more frequently in XY systems, while it is comparably rare in ZW species [25,43]. This discrepancy is unrelated to the evolutionary age of the sex chromosome system [44], and models indicate that it could rather be a consequence of stronger sexual selection in males, which is the heterogametic sex in XY systems [45].

An expression disequilibrium of X/Z-linked genes cannot only occur in the cases where chromosome-wide DC is absent. Some mammalian genes referred to as escapees, for example, the Histone demethylase gene *Kdm6a*, may get expressed from the inactivated female X. Predominantly, such escape is not constitutive, but depends on developmental stage, tissue type, or genetic background of the individual [46] and appears to be much more prevalent in humans compared to, e.g., mice [47]. Since the second, inactive X is only present in females, one could regard it as an additional “reservoir” providing a potential advantage with regard to allelic diversity and gene dosage [48]. Understanding phenotypic consequences of mutations in genes that can escape X chromosome inactivation is therefore an important field of research [49] highlighting the significance of sex chromosome dosage balancing in physiology and disease.

### Gene expression changes upon autosomal aneuploidy

Is there a DC mechanism akin to the X chromosome when autosomes become aneuploid [50]? In human fibroblasts obtained from individuals with trisomy of chromosome 21, 13, and 18, mRNA and protein levels are typically increased to the expected value of 1.5-fold [51]. Similar effects are observed in fibroblasts obtained from mice with Robertsonian translocations, where gene expression increases proportional to gene copy number [52]. By contrast, segmental aneuploidies in *Drosophila* were reported to experience DC [53,54]. Nonetheless, compensation does not always appear to be complete, as a 2-fold change in DNA typically results in about a 1.5-fold increase in gene expression [55,56]. *Saccharomyces cerevisiae*, in turn, seems again similar to mammals, where gene expression differences correspondingly scale with the affected chromosome and are not compensated [10,57]. This is also reflected at the proteome level [58], where, however, some protein level buffering counteracting the gene dosage alteration can be detected [21]. Autosomal aneuploidies can also impact the expression of other genes in *trans*, for example, if the region originally affected by a copy number change harbors a transcription factor or a microRNA gene [59]. A study in maize found that *cis*-specific up-regulation caused by a segmental trisomy occurs in a tissue invariant fashion, while *trans*-effects appeared rather tissue specific [18]. One aspect to consider for these seemingly distinct outcomes concerning aneuploidy-induced DC might be that such a mechanism needs time to be established on affected chromosomes after the alteration has occurred. The expression data for segmental aneuploidies have been generated from adult flies [53,54], and, hence, its establishment along embryogenesis and the larval stages remains unclear. Furthermore, different yeast cells display a great degree of nongenetic phenotypic variability in cellular responses right after the induction of the very same chromosomal alteration, which is also an intriguing phenotypic manifestation of mice with Robertsonian translocations [21,58]. Variability might be an instant response, which then might get canalized into a more stable form of autosomal DC. If a “noncanonical” form of DC indeed exists for autosomes, it will be interesting to unravel similarities and differences to the features acting on the X-chromosome: How are the affected chromosomes and genes recognized and distinguished from the euploid population? Are DC mechanisms acting on genes individually, or does a universal regulation occur at a chromosome-wide scale, irrespective of the nature or function of the single gene? In the absence of a universal “autosome-DC” pathway, feedback mechanisms could provide a means for how subtle expression perturbations could be propagated into regulatory networks [60,61]. Another interesting aspect of autosomal DC is how precision for an approximately 2-fold effect could be achieved. Addressing such questions could be key to understanding the diverse facets of aneuploidy during development and disease.

### Dosage alterations at the individual gene level

Dosage alterations do not only occur chromosome wide but also at the other end of the spectrum: the individual gene level (Fig 1). Recent efforts by human exome and genome sequencing consortia probably provide the most compelling view on this aspect in a natural population. The Exome Aggregation Consortium (ExAC) and Genome Aggregation Database (gnomAD) studies found that the human genome displays a high degree of selection against nearly all classes of structural variants affecting single genes, with the interesting exception of copy gains [62]. The latter finding is in agreement with gene duplication events being a key process in evolution (Box 2) [63,64], although they can be sometimes associated with negative phenotypic consequences. A single *Drosophila* locus (*Tpl*) causes lethality when present in either 3 copies or a single copy [11,65]. In humans, duplications and heterozygous deletions of the cohesin loading factor *NIPBL* lead to intellectual disability, developmental delay, craniofacial defects, and limb malformations [66,67]. By integrating information on copy numbers in 753,994 human individuals, a recent study produced a more comprehensive dataset including a statistical model to predict dosage sensitivity, which defined 3,006 haploinsufficient and 295 triplosensitive genes [68]. Thus, single copy gains are not always strongly deleterious, and its effects appear generally less pronounced and subtle compared to the corresponding gene losses. This is in agreement with the stoichiometric perturbations upon gene duplication (3:2 copies) being less pronounced than upon deletion (1:2 copies).

**Box 2. Gene-by-gene dosage alterations and evolution**

Gene duplications are crucial to the evolution of novel genes and regulatory elements, because they create redundancy, which liberates DNA sequences from previous selective constraints. Duplicated copies, whereupon they are referred to as gene paralogues, can arise by (1) tandem and segmental duplication upon DNA rearrangements; (2) retrotransposition; and (3) whole genome duplication [116]. Whole genome duplications are observed several times throughout eukaryotic evolution and provide a balanced increase in ploidy. They are typically unstable and followed by widespread gene loss, for example, by obtaining damaging mutations referred to as pseudogenization [117]. Paralogues that are maintained from whole genome duplication receive a special name, so-called Ohnologues [64]. Examples of paralogue functions can be found in almost any species and virtually all aspects of biology. Human-specific gene duplication events coincide with and were proposed to be causally involved in the beginning of the neocortex expansion [118]. Compared to animals, plants have comparably higher duplication rates. Polyploidy as well as the retention of stress response genes may facilitate their rapid adaptation to changing environments [119–121].

From a dosage point of view, the counterpart of beneficial gene duplications are natural examples of monoallelism, which induce functional hemizygosity at the gene-by-gene level. There are 2 major categories of monoallelism: (1) genetic, where changes to the primary DNA sequence result in only a single allele being able to make a functional protein; and (2) epigenetic, where both parental alleles are present and per se functional, but only one is transcribed, whereas the other one is silenced. Allelic exclusion belongs to the first category and allows only a single type of antigen receptor genes to be expressed in B cells and T cells. Nonfunctional *Ig* and *TCR* alleles are created by incomplete or nonproductive V(D)J recombination (Fig 1). The phenomenon of genomic imprinting belongs to the second category of “epigenetic monoallelism.” For imprinted genes, the allele choice is nonrandom and occurs in a parent of origin–specific manner [122]. A well-studied example is the *IGF2* gene, which is exclusively expressed from the paternal genome and is important for fetal growth and development of various tissues such as the placenta or the brain [123]. Monoallelic expression is also found in olfactory sensory neurons, where only a single, but in this case randomly chosen, olfactory receptor (OR) gene is expressed. The monogenic and monoallelic expression enables defined olfactory perception of odors in the olfactory epithelium [124]. Cells in the immune and nervous system appear to employ monoallelism to ensure a high degree of cellular diversity and individuality. Although random monoallelic expression was described to occur on other autosomal genes, the functional relevance of this phenomenon could not be unambiguously resolved to date [125–129].

Heterozygous mutations can sometimes produce detrimental gain-of-function alleles, but when they reduce gene function and cause an associated decrease in organismal fitness, this is referred to as haploinsufficiency. One of the first systematic characterizations of haploinsufficiency was conducted in *Drosophila*, which revealed a few dozen *Minute* loci. In heterozygosity, they display aberrant bristles, compromised viability and fertility as well as altered developmental timing [69]. Such developmental phenotypes caused by heterozygous variants are also prevalent in humans, where they frequently lead to rare disorders [4]. Based on the probability of genetic variation obtained in ExAC and gnomAD, the fraction of human haploinsufficient genes is estimated to reach approximately 20% [5]. Using the yeast knockout collection, it was found that heterozygous deletion of about 3% of the budding yeast genes respond with defects in cell proliferation in optimal growth conditions [70], but this fraction increases to 20% in different growth conditions [71]. Interestingly, when morphological features are analyzed, 75% of essential genes in yeast showed a phenotype in heterozygosity [72]. Thus, haploinsufficiency is apparently context dependent and (not only in yeast) influenced by environmental conditions or nutrient supply. This is further complicated by the fact that most haploinsufficiency phenotypes are associated with variable expressivity (i.e., the phenotypes differ among affected individuals) and show reduced penetrance (i.e., some individuals show phenotypes, while others are seemingly unaffected) and, hence, can be difficult to detect and study.

**Fig 3:**
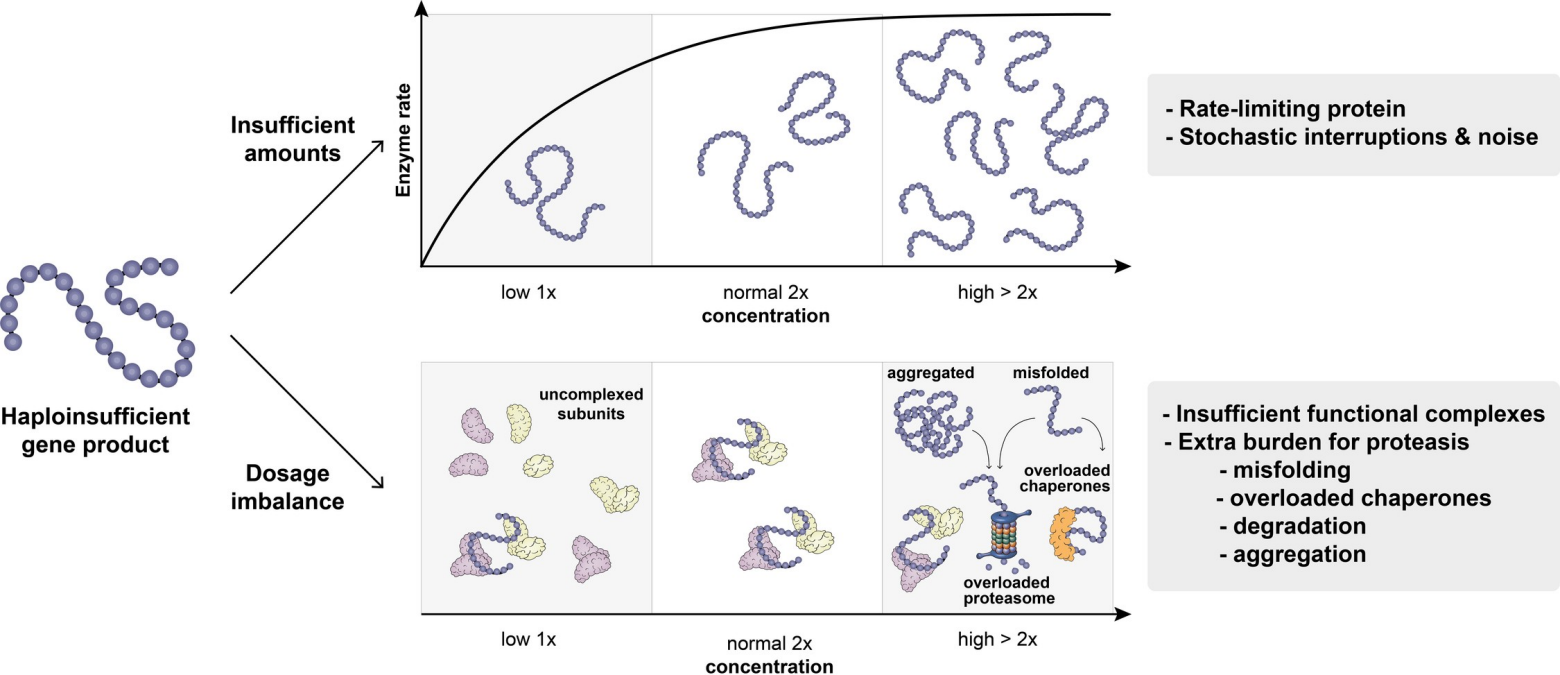
Molecular consequences upon gene dosage alterations. If gene dosage alterations reduce gene quantity, insufficient amounts of a protein (blue chain) can be produced (top). If the affected protein is rate limiting, for example, an enzyme, this can lead to fitness defects. Increases and reductions in gene quantity can also result in disrupted stoichiometry (bottom), if the affected protein (blue chain) interacts with other cellular macromolecules (illustrated as a complex consisting of a dimeric subunit A (purple), a dimeric subunit C (yellow) and the affected protein functioning as a bridge subunit B (blue)). This can lead to (a) change in the amount of functional complexes; (b) aggregation of uncomplexed subunits; and (c) overload of the chaperones and proteostasis network. Copy number increases can also lead to the aggregation of the protein products simply by reaching a critical, abnormally high concentration, promoting pathological transitions due to the protein’s physical properties.

What are the common attributes of such genes where dosage appears to be so critical? One immediate effect of hemizygosity is that (in absence of a buffering mechanism, see below) lower amounts are being produced, which may be important where the gene product is rate limiting (Fig 3). Accordingly, some yeast haploinsufficiency genes were found to function in metabolic processes, and these effects can be alleviated by slowing the growth rate in minimal media [70]. Expression from a single allele, instead of two, was also proposed to increase transcription noise and cause stochastic delays and interruptions of gene expression [73,74]. Another explanation is not the reduced expression per se but alterations in stoichiometry [75,76]. Dosage-sensitive genes are generally more likely to be found in multisubunit protein complexes [77], and, indeed, many of the *Minute* phenotypes were found to be caused by loss-of-function alleles for ribosomal proteins [78]. Under this dosage balance hypothesis, both reduction as well as overexpression of a given gene are expected to lead to fitness defects, and rebalancing the expression of the interaction partners should alleviate such effects. For example, lethality by expressing an extra copy of beta tubulin can be rescued by providing an extra copy of alpha tubulin [79]. A recent study has analyzed the contribution of these mechanisms more systematically and investigated the effects of single-copy losses and gains of each yeast gene. This brought forward that there is no single, universal explanation for haploinsufficiency, and all of the aforementioned possibilities contribute to the organismal fitness defects [80]. In the light of the striking prevalence of haploinsufficiency in humans [5,6], studying gene dosage in the context of cellular morphology [72] and from a multicellular point of view is a pressing issue in order to understand its relevance for genome evolution and disease manifestation.

### Robustness and buffering between alleles and paralogues

For human genetic disorders, understanding the cross talk between alleles and paralogous genes (Box 2) is of particular interest, because unlocking their potential could provide a compensatory mechanism for genetic or environmental perturbations. For example, CRISPR-mediated activation of the remaining functional copy can rescue obesity caused by *Sim1* haploinsufficiency in mice [81]. Compensatory up-regulation can also be intrinsic and a consequence of genetic robustness, i.e., the capacity of an organism to withstand harmful mutations [82]. The Resilience Project, for example, uncovered several human individuals with mutations in Mendelian disease genes, which surprisingly did not show any of the severe clinical symptoms commonly associated with that particular mutation [83]. In the case of a heterozygous mutation, an obvious mechanism is the up-regulation of the unaffected allele. In *Nipbl+/-* mutant mice, Nipbl transcript levels are only reduced by 25% to 30% instead of 50%, indicative of a compensatory up-regulation from the *wild-type* allele. This compensation is thought to contribute to the degree and severity of phenotype manifestation [84].

How can such regulatory loops work at the molecular level? Complex autoregulatory and cross-regulatory loops occur, for example, in the genes encoding proteins functioning in RNA splicing such as the SR genes. Those undergo alternative splicing, where productive splicing results in a full-length SR protein to be produced, while inclusion of an early in-frame stop codon-containing “poison exon” triggers RNA decay [85]. Thereby, protein level alterations of spliceosomal components and regulatory proteins affect the production of alternative splice variants from their own corresponding genes, and this ensures maintaining their appropriate levels. Another form of responsiveness and cross talk that can occur upon gene mutation is the acquisition of secondary mutations. In a yeast study, the independent knockout of the same gene was shown to frequently evolve mutations in the very same secondary gene [86].

**Fig 4:**
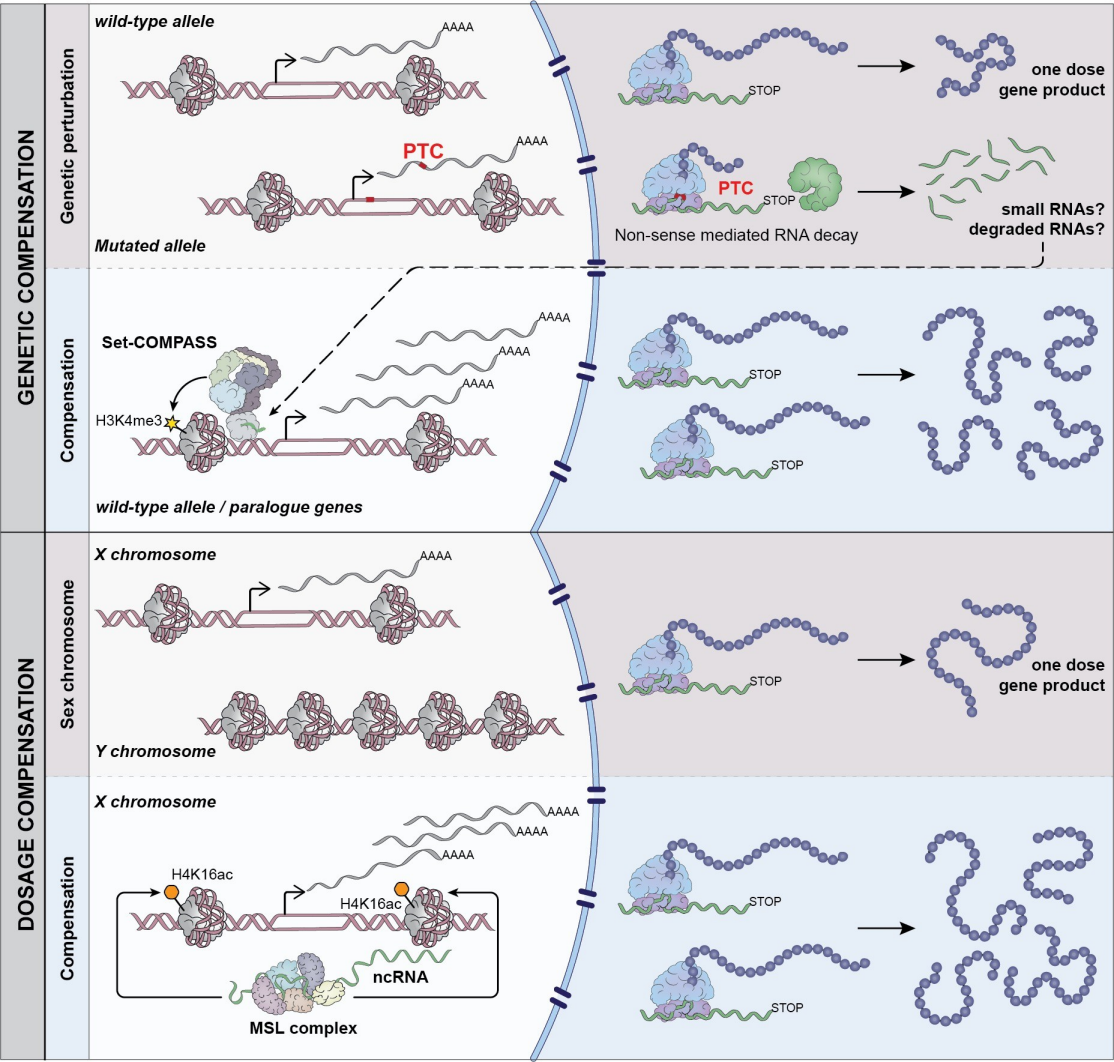
Comparison of genetic compensation and X chromosome DC. The illustration shows the genetic compensation mechanism, where a premature termination codon in the DNA, produces an RNA, which is exported to the cytoplasm. Upon translation, the premature termination codon-containing transcript is recognized as “faulty” by the NMD pathway and degraded. It is assumed that the degradation products (small RNAs?) can signal back to the corresponding locus in the nucleus, which promotes an increase of Histone H3 Lysine 4 trimethylation (H3K4me3) at the promoter of the (a) unaffected allele; and (b) of gene paralogues that have sequence similarity to the original locus. This can then enhance transcription, which functionally rescues the effects of the original mutation. In *Drosophila* DC, noncoding RNAs are produced in cis from the hemizygous X chromosome in males. This induces the recruitment of the MSL2 protein of the MSL complex. The MSL complex acetylates Histone H4 Lysine 16 (H4K16ac), which triggers the chromatin decompaction and promotes transcription of expressed genes on the X. DC, dosage compensation; NMD, nonsense mediated decay.

More recently, the exciting finding was made that mutant mRNA degradation can trigger a more general cellular response upon genetic perturbations [87,88]. This mechanism is referred to as genetic compensation and provides a form of robustness, which differs from the aforementioned examples. It is a general mechanism that occurs upstream of the protein product, i.e., is independent of the protein sequence or function the affected gene encodes. With these features, it is conceptually similar to the X chromosome DC mechanism discussed above (Fig 4).

Genetic compensation occurs for transcripts harboring a premature termination codon. This can trigger the up-regulation of the remaining allele and paralogous genes that can functionally substitute for the defective copy. Up-regulation is dependent on the nonsense-mediated decay (NMD) pathway and is associated with the enrichment of histone H3 Lysine 4 trimethylation at the promoter regions of the compensatory genes. This pathway exists in at least mouse [87], zebrafish [89], and *C*. *elegans* [90] and may provide an explanation for the fact that incomplete penetrance of genetic mutations is relatively common [91].

Genetic compensation may be triggered by small RNA degradation products, which could serve as guide molecules and target chromatin-modifying complexes by complementary base pairing to nascent transcripts of the affected gene loci [92,93]. Another possibility is that a response similar to the RNAa mechanism is elicited, where promoter-targeted microRNAs (miRNAs) activate transcription [94]. Furthermore, pairing of homologous sequences and chromosomes may be another mechanism through which alleles would be able to “cross talk.” An inherent feature of the NMD pathway is that it requires translation and depends on the position of a premature termination codon with regard to the exon junction. Hence, genetic compensation is unable to be triggered from complete deletions or duplicates created by retrotransposition, since they are created via a cDNA intermediate where the exon–intron structure is lost. The prevalence of genetic compensation triggered by premature termination codons and its importance for the manifestation of DD in humans remain unexplored to date, but it may be one explanation for the reduced penetrance and variable expressivity of haploinsufficient genes.

### Gene dosage alterations and developmental disorders

The realization that gene dosage alterations can be linked to a human phenotypic abnormality dates back to 1959, where trisomy of chromosome 21 was found to be the underlying cause of Down syndrome (DS) [95]. Trisomic mouse models have helped to understand the gene dosage aspects of DS and its etiology. Together with molecular analysis of human samples, they allowed the identification of genes, whose overexpression have been associated with the mental retardation (*DYRK1A*), heart defects (*COL6A1*), leukemia (*ETS2*), and the premature aging and neurodegenerative features (*SOD1* and *APP*) of the disease, respectively. Trisomy of chromosome 18 (Edwards syndrome) and 13 (Patau syndrome) are the only other autosomal aneuploidies compatible with live births, but the median life expectancy is below two weeks, while survival beyond the first year of life is rare and often attributed to mosaicism [96,97]. Sex chromosomal aneuploidies, also referred to as gonosomal aneuploidies, are compatible with survival to adulthood. Interestingly, the majority of XXX individuals display hardly any symptoms. Turner syndrome (X0 females) or Klinefelter syndrome (XXY males) causes sterility, cognitive, and neurobehavioral symptoms. It is thought that the abnormally low or high expression of X chromosome escapees (see above) contribute to the symptoms associated with these latter 2 syndromes [98]. It is noteworthy that other aneuploidies lead to embryo death during development, but are pretty prevalent and not detrimental before implantation [99,100]. In chimeric mouse embryos, aneuploid cells are progressively eliminated by apoptosis and compromised proliferation only after implantation [101]. Interindividual variability is a confounding factor in the analysis of aneuploid human cells, but a recent study revealed some general cellular and molecular responses upon Trisomy including nuclear abnormalities and alterations in lipid levels [51]. How those relate to the postimplantation developmental failures remains to be elucidated.

The diagnostics of such larger gene dosage alterations have a long history thanks to karyotyping or fluorescence in situ hybridization approaches. The technological advances of the last decade have now enabled NGS to become part of standard medical diagnostics, which led to a significant increase in the number of single gene mutations associated with DD [4]. The Online Mendelian Inheritance in Man (OMIM [102]) database contains information on 4,146 single gene disorders associated with distinct phenotypes, many of which are of pediatric, developmental onset. The DDD project is an exemplary study providing exomes of 4,293 families with affected individuals, which revealed 94 single disease genes with damaging de novo mutations. The majority (60%) of heterozygous *de novo* mutations in DD genes induce a loss of function in the encoded protein, indicating that haploinsufficiency is a major mechanism underlying DDs [4]. Conversely, recessive coding variants only account for a small fraction of DD cases [103]. In light of the highly overlapping symptoms and low clinical recognizability of some DDs [104], it is possible that gene dosage imbalance caused by heterozygous mutations, similar to the aneuploidies, induces common cellular perturbations. Patient-derived cells, induced pluripotent stem cells, and organoids are powerful systems for analyzing the molecular mechanisms underlying such syndromes. A landmark study in the field generated brain organoids of a patient with microcephaly caused by mutations in *CDK5RAP2*. The organoids recapitulated features of the neurodevelopmental disorder by, for example, displaying aberrant neuroepithelia and signs of premature neural differentiation [105].

The existence of a gene duplicate can, in turn, be exploited for the treatment of disorders caused by mutations in single genes. For example, *SMN2* is a paralogue of the *SMN1* gene and this duplicated copy is only present in humans, but not other primates [106]. SMN1 mutations cause spinal muscular atrophy, a severe disease associated with a progressive loss of motoneurons. The presence of the *SMN2* paralogue was successfully harnessed to treat spinal muscular atrophy: Because of a noncoding point mutation, the *SMN2* mRNA splicing pattern typically lacks exon 7 and produces a truncated protein. This truncated protein produced from *SMN2* is unable to complement the lack of SMN protein in patients. However, treatment with an antisense oligonucleotide, now approved under the name Nusinersen (Spinraza), can promote exon 7 inclusion. This leads to the production of full-length SMN protein from SMN2, which alleviates the spinal muscular atrophy symptoms due to lack of protein produced from *SMN1* [107].

## Concluding remarks

Gene dosage alterations pose a challenge to organisms: They need to juggle the benefit of remaining evolvable with the deleterious potential of the associated expression alterations. Studies of the X chromosome, as well as systems biology approaches in model organisms such as yeast, have uncovered common principles acting on aneuploid chromosomes. Nonetheless, chromosome-wide DC is not as universal in nature as previously thought, at least when assayed at the RNA level. Probing the proteome for DC is conceptually an important problem that will need to be addressed in future research. If lack of DC is indeed common, it will be interesting to explore the reasons why certain organisms tolerate aneuploidy, dosage imbalance, and the associated cellular consequences better than others. Some factors that may need to be considered in that regard are gene content and location of dosage sensitive genes, genetic networks and buffering, specific form and challenges of organismal development, aneuploidy sensitivity of different cell types and organs, as well as ecological constraints. Chromatin-centered characterizations performed in nonmodel organisms may, in turn, be promising to unravel entirely new gene regulatory modules and other forms of how compensation can be achieved. Identifying such mechanisms will also help to understand the regulatory mechanisms on haploinsufficiency genes, particularly in multicellular contexts. For this, the advances in CRISPR screening, high content imaging, and artificial intelligence–aided computation will be a fruitful way forward, where phenotype discovery could, for example, focus on cellular shape or communication.

In light of the compelling data provided by human genome and exome sequencing efforts, one needs to keep in mind that the human population is not necessarily comparable to studies performed with inbred model organisms in laboratory conditions. Instead, second site mutations, epistasis, the environment, or microbiome can contribute to the highly variable phenotypes of heterozygous mutations and associated disorders. Besides the identification of distinctive phenotypic features, diagnosis by NGS, together with the stratification by patient networks, has an enormous potential to change the perspectives for individuals affected by rare genetic diseases and for the design of therapeutic protocols. The identification and the targeting of common molecular and cellular features and their relation to systemic fitness defects may be a more immediate alternative to overcome the challenges associated with personalized therapies. Instead of a single-gene centric view, we envision that the future will lie in systems-based and complex interaction network approaches to understand and ameliorate the life of patients affected by haploinsufficiency syndromes. The large-scale sequencing efforts of the healthy population, in turn, can aid in providing insights into potential resilience mechanisms, for example, the ones operating via the recently described genetic compensation pathway. In summary, the ever-growing knowledge of the human genome allows an unprecedented possibility to integrate evolutionary concepts into our investigations of human diseases caused by gene dosage alterations.

**Key Learning Points**

- Gene dosage alterations are a key requirement for evolution, but can carry a pathogenic potential.
- Studying regulatory mechanisms on sex chromosomes and the evolutionary diversity of such pathways may provide insights into diseases caused by dosage changes.
- Humans contain a surprisingly high fraction of genes that are highly intolerant to heterozygous mutation.
- Dosage changes do not only induce gene-specific, but also common cellular effects associated with aggregation, proteotoxicity, and a general stress response.
- Compensatory mechanisms do not only occur on sex chromosomes, but also on autosomes—from the individual up to the chromosome-wide level.

**Top Five Papers**

1. Karczewski KJ, Francioli LC, Tiao G, Cummings BB, Alföldi J, Wang Q, et al. The mutational constraint spectrum quantified from variation in 141,456 humans. Nature. 2020;581:434–443.
2. El-Brolosy MA, Kontarakis Z, Rossi A, Kuenne C, Günther S, Fukuda N, et al. Genetic compensation triggered by mutant mRNA degradation. Nature. 2019;568:193–197.
3. Beach RR, Ricci-Tam C, Brennan CM, Moomau CA, Hsu PH, Hua B, et al. Aneuploidy Causes Non-genetic Individuality. Cell. 2017;169:229–242 e21.
4. Bellott DW, Page DC. Dosage-sensitive functions in embryonic development drove the survival of genes on sex-specific chromosomes in snakes, birds, and mammals. Genome Res. 2021. doi: 10.1101/gr.268516.120
5. Lee H, Cho D-Y, Whitworth C, Eisman R, Phelps M, Roote J, et al. Effects of Gene Dose, Chromatin, and Network Topology on Expression in Drosophila melanogaster. PLoS Genet. 2016;12:e1006295.

## Supporting information

S1 File Species, sex chromosome system, mode of DC, and references related to evolutionary tree in Fig 2. DC, dosage compensation. (PDF)

## References

[1] Siegel JJ, Amon A. New insights into the troubles of aneuploidy. Annu Rev Cell Dev Biol. 2012;28:189–214. doi: 10.1146/annurev-cellbio-101011-155807 22804579 PMC3919630

[2] Van de Peer Y, Mizrachi E, Marchal K. The evolutionary significance of polyploidy. Nat Rev Genet. 2017;18:411–424. doi: 10.1038/nrg.2017.26 28502977

[3] Samata M, Akhtar A. Dosage Compensation of the X Chromosome: A Complex Epigenetic Assignment Involving Chromatin Regulators and Long Noncoding RNAs. Annu Rev Biochem. 2018;87:323–350. doi: 10.1146/annurev-biochem-062917-011816 29668306

[4] Deciphering Developmental Disorders Study. Prevalence and architecture of de novo mutations in developmental disorders. Nature. 2017;542:433–438. doi: 10.1038/nature21062 28135719 PMC6016744

[5] Karczewski KJ, Francioli LC, Tiao G, Cummings BB, Alföldi J, Wang Q, et al. The mutational constraint spectrum quantified from variation in 141,456 humans. Nature. 2020;581:434–443. doi: 10.1038/s41586-020-2308-7 32461654 PMC7334197

[6] Lek M, Karczewski KJ, Minikel EV, Samocha KE, Banks E, Fennell T, et al. Analysis of protein-coding genetic variation in 60,706 humans. Nature. 2016;536:285–291. doi: 10.1038/nature19057 27535533 PMC5018207

[7] Vasudevan A, Schukken KM, Sausville EL, Girish V, Adebambo OA, Sheltzer JM. Aneuploidy as a promoter and suppressor of malignant growth. Nat Rev Cancer. 2021;21:89–103. doi: 10.1038/s41568-020-00321-1 33432169

[8] Santaguida S, Amon A. Short- and long-term effects of chromosome mis-segregation and aneuploidy. Nat Rev Mol Cell Biol. 2015;16:473–485. doi: 10.1038/nrm4025 26204159

[9] Blakeslee AF, Belling J, Farnham ME. Chromosomal duplication and Mendelian phenomena in Datura mutants. Science. 1920;52:388–390. doi: 10.1126/science.52.1347.388 17829955

[10] Torres EM, Sokolsky T, Tucker CM, Chan LY, Boselli M, Dunham MJ, et al. Effects of aneuploidy on cellular physiology and cell division in haploid yeast. Science. 2007;317:916–924. doi: 10.1126/science.1142210 17702937

[11] Lindsley DL, Sandler L, Baker BS, Carpenter AT, Denell RE, Hall JC, et al. Segmental aneuploidy and the genetic gross structure of the Drosophila genome. Genetics. 1972;71:157–184. doi: 10.1093/genetics/71.1.157 4624779 PMC1212769

[12] Lee EA, Darrah LL, Coe EH. Dosage effects on morphological and quantitative traits in maize aneuploids. Genome. 1996;39:898–908. doi: 10.1139/g96-113 18469943

[13] García-Bellido A, del Prado JM, Botas J. The effect of aneuploidy on embryonic development in Drosophila melanogaster. Mol Gen Genet. 1983;192:253–263.

[14] Mirkovic M, Guilgur LG, Tavares A, Passagem-Santos D, Oliveira RA. Induced aneuploidy in neural stem cells triggers a delayed stress response and impairs adult life span in flies. PLoS Biol. 2019;17:e3000016. doi: 10.1371/journal.pbio.3000016 30794535 PMC6402706

[15] Resende LP, Monteiro A, Brás R, Lopes T, Sunkel CE. Aneuploidy in intestinal stem cells promotes gut dysplasia in Drosophila. J Cell Biol. 2018;217:3930–3946. doi: 10.1083/jcb.201804205 30282810 PMC6219720

[16] Clemente-Ruiz M, Murillo-Maldonado JM, Benhra N, Barrio L, Perez L, Quiroga G, et al. Gene Dosage Imbalance Contributes to Chromosomal Instability-Induced Tumorigenesis. Dev Cell. 2016;36:290–302. doi: 10.1016/j.devcel.2016.01.008 26859353

[17] Longo L, Bygrave A, Grosveld FG, Pandolfi PP. The chromosome make-up of mouse embryonic stem cells is predictive of somatic and germ cell chimaerism. Transgenic Res. 1997;6:321–328. doi: 10.1023/a:1018418914106 9322369

[18] Makarevitch I, Harris C. Aneuploidy causes tissue-specific qualitative changes in global gene expression patterns in maize. Plant Physiol. 2010;152:927–938. doi: 10.1104/pp.109.150466 20018594 PMC2815861

[19] Henry IM, Dilkes BP, Miller ES, Burkart-Waco D, Comai L. Phenotypic consequences of aneuploidy in Arabidopsis thaliana. Genetics. 2010;186:1231–1245. doi: 10.1534/genetics.110.121079 20876566 PMC2998307

[20] Tsai H-J, Nelliat AR, Choudhury MI, Kucharavy A, Bradford WD, Cook ME, et al. Hypo-osmotic-like stress underlies general cellular defects of aneuploidy. Nature. 2019;570:117–121. doi: 10.1038/s41586-019-1187-2 31068692 PMC6583789

[21] Beach RR, Ricci-Tam C, Brennan CM, Moomau CA, Hsu PH, Hua B, et al. Aneuploidy Causes Non-genetic Individuality. Cell. 2017;169:229–242 e21. doi: 10.1016/j.cell.2017.03.021 28388408 PMC5441241

[22] Rehen SK, McConnell MJ, Kaushal D, Kingsbury MA, Yang AH, Chun J. Chromosomal variation in neurons of the developing and adult mammalian nervous system. Proc Natl Acad Sci U S A. 2001;98:13361–13366. doi: 10.1073/pnas.231487398 11698687 PMC60876

[23] Charlesworth B, Charlesworth D. The degeneration of Y chromosomes. Philos Trans R Soc Lond B Biol Sci. 2000;355:1563–1572. doi: 10.1098/rstb.2000.0717 11127901 PMC1692900

[24] Ohno S. Sex Chromosomes and Sex-linked Genes. Springer, Berlin, Heidelberg; 1966.

[25] Gu L, Walters JR. Evolution of Sex Chromosome Dosage Compensation in Animals: A Beautiful Theory, Undermined by Facts and Bedeviled by Details. Genome Biol Evol. 2017;9:2461–2476. doi: 10.1093/gbe/evx154 28961969 PMC5737844

[26] Galupa R, Heard E. X-Chromosome Inactivation: A Crossroads Between Chromosome Architecture and Gene Regulation. Annu Rev Genet. 2018;52:535–566. doi: 10.1146/annurev-genet-120116-024611 30256677

[27] Strome S, Kelly WG, Ercan S, Lieb JD. Regulation of the X chromosomes in Caenorhabditis elegans. Cold Spring Harb Perspect Biol. 2014;6. doi: 10.1101/cshperspect.a018366 24591522 PMC3942922

[28] Dawes HE, Berlin DS, Lapidus DM, Nusbaum C, Davis TL, Meyer BJ. Dosage compensation proteins targeted to X chromosomes by a determinant of hermaphrodite fate. Science. 1999; 1800–1804. doi: 10.1126/science.284.5421.1800 10364546

[29] Belote JM, Lucchesi JC. Male-specific lethal mutations of Drosophila melanogaster. Genetics. 1980;96:165–186. doi: 10.1093/genetics/96.1.165 6781985 PMC1214287

[30] Marahrens Y, Panning B, Dausman J, Strauss W, Jaenisch R. Xist-deficient mice are defective in dosage compensation but not spermatogenesis. Genes Dev. 1997;11:156–166. doi: 10.1101/gad.11.2.156 9009199

[31] Yang L, Kirby JE, Sunwoo H, Lee JT. Female mice lacking Xist RNA show partial dosage compensation and survive to term. Genes Dev. 2016;30:1747–1760. doi: 10.1101/gad.281162.116 27542829 PMC5002979

[32] Muyle A, Zemp N, Deschamps C, Mousset S, Widmer A, Marais GAB. Rapid de novo evolution of X chromosome dosage compensation in Silene latifolia, a plant with young sex chromosomes. PLoS Biol. 2012;10:e1001308. doi: 10.1371/journal.pbio.1001308 22529744 PMC3328428

[33] Keller Valsecchi CI, Marois E, Basilicata MF, Georgiev P, Akhtar A. Distinct mechanisms mediate X chromosome dosage compensation in Anopheles and Drosophila. Life Sci Alliance. 2021;4. doi: 10.26508/lsa.202000996 34266874 PMC8321682

[34] Rovatsos M, Kratochvíl L. Evolution of dosage compensation does not depend on genomic background. Mol Ecol. 2021;30:1836–1845. doi: 10.1111/mec.15853 33606326

[35] Picard MAL, Vicoso B, Roquis D, Bulla I, Augusto RC, Arancibia N, et al. Dosage Compensation throughout the Schistosoma mansoni Lifecycle: Specific Chromatin Landscape of the Z Chromosome. Genome Biol Evol. 2019;11:1909–1922. doi: 10.1093/gbe/evz133 31273378 PMC6628874

[36] Bista B, Wu Z, Literman R, Valenzuela N. Thermosensitive sex chromosome dosage compensation in ZZ/ZW softshell turtles, Apalone spinifera. Philos Trans R Soc Lond B Biol Sci. 2021;376:20200101. doi: 10.1098/rstb.2020.0101 34304598 PMC8310717

[37] Furman BLS, Metzger DCH, Darolti I, Wright AE, Sandkam BA, Almeida P, et al. Sex Chromosome Evolution: So Many Exceptions to the Rules. Genome Biol Evol. 2020;12:750–763. doi: 10.1093/gbe/evaa081 32315410 PMC7268786

[38] Zhang SO, Mathur S, Hattem G, Tassy O, Pourquié O. Sex-dimorphic gene expression and ineffective dosage compensation of Z-linked genes in gastrulating chicken embryos. BMC Genomics. 2010;11:13. doi: 10.1186/1471-2164-11-13 20055996 PMC2821371

[39] Zimmer F, Harrison PW, Dessimoz C, Mank JE. Compensation of Dosage-Sensitive Genes on the Chicken Z Chromosome. Genome Biol Evol. 2016;8:1233–1242. doi: 10.1093/gbe/evw075 27044516 PMC4860703

[40] Vicoso B, Emerson JJ, Zektser Y, Mahajan S, Bachtrog D. Comparative sex chromosome genomics in snakes: differentiation, evolutionary strata, and lack of global dosage compensation. PLoS Biol. 2013;11:e1001643. doi: 10.1371/journal.pbio.1001643 24015111 PMC3754893

[41] Bellott DW, Page DC. Dosage-sensitive functions in embryonic development drove the survival of genes on sex-specific chromosomes in snakes, birds, and mammals. Genome Res. 2021. doi: 10.1101/gr.268516.120 33479023 PMC7849413

[42] White MA, Kitano J, Peichel CL. Purifying Selection Maintains Dosage-Sensitive Genes during Degeneration of the Threespine Stickleback Y Chromosome. Mol Biol Evol. 2015;32:1981–1995. doi: 10.1093/molbev/msv078 25818858 PMC4635650

[43] Mank JE. Sex chromosome dosage compensation: definitely not for everyone. Trends Genet. 2013;29:677–683. doi: 10.1016/j.tig.2013.07.005 23953923

[44] Metzger DCH, Sandkam BA, Darolti I, Mank JE. Rapid Evolution of Complete Dosage Compensation in Poecilia. Genome Biol Evol. 2021;13. doi: 10.1093/gbe/evab155 34240180 PMC8325565

[45] Mullon C, Wright AE, Reuter M, Pomiankowski A, Mank JE. Evolution of dosage compensation under sexual selection differs between X and Z chromosomes. Nat Commun. 2015;6:7720. doi: 10.1038/ncomms8720 26212613 PMC4525201

[46] Andergassen D, Dotter CP, Wenzel D, Sigl V, Bammer PC, Muckenhuber M, et al. Mapping the mouse Allelome reveals tissue-specific regulation of allelic expression. Elife. 2017;6. doi: 10.7554/eLife.25125 28806168 PMC5555720

[47] Tukiainen T, Villani A-C, Yen A, Rivas MA, Marshall JL, Satija R, et al. Landscape of X chromosome inactivation across human tissues. Nature. 2017;550:244–248. doi: 10.1038/nature24265 29022598 PMC5685192

[48] Posynick BJ, Brown CJ. Escape From X-Chromosome Inactivation: An Evolutionary Perspective. Front Cell Dev Biol. 2019;7:241. doi: 10.3389/fcell.2019.00241 31696116 PMC6817483

[49] Basilicata MF, Bruel A-L, Semplicio G, Valsecchi CIK, Aktaş T, Duffourd Y, et al. De novo mutations in MSL3 cause an X-linked syndrome marked by impaired histone H4 lysine 16 acetylation. Nat Genet. 2018;50:1442–1451. doi: 10.1038/s41588-018-0220-y 30224647 PMC7398719

[50] Kojima S, Cimini D. Aneuploidy and gene expression: is there dosage compensation? Epigenomics. 2019;11:1827–1837. doi: 10.2217/epi-2019-0135 31755744 PMC7132608

[51] Hwang S, Cavaliere P, Li R, Zhu LJ, Dephoure N, Torres EM. Consequences of aneuploidy in human fibroblasts with trisomy 21. Proc Natl Acad Sci U S A. 2021;118(6):e2014723118. doi: 10.1073/pnas.2014723118 33526671 PMC8017964

[52] Williams BR, Prabhu VR, Hunter KE, Glazier CM. Aneuploidy affects proliferation and spontaneous immortalization in mammalian cells. Science. 2008;322(5902):703–9 https://science.sciencemag.org/content/322/5902/703.abstract?casa_token=Br3wYEq5FxUAAAAA:3gRdKN2goYTa7BSzownjgt3fo4XSqMk1SvirxjTETQ2vEaGjJuP66qx30lNaKXC2z_ys0wVNCPJG_asu doi: 10.1126/science.1160058 18974345 PMC2701511

[53] Stenberg P, Lundberg LE, Johansson A-M, Rydén P, Svensson MJ, Larsson J. Buffering of segmental and chromosomal aneuploidies in Drosophila melanogaster. PLoS Genet. 2009;5:e1000465. doi: 10.1371/journal.pgen.1000465 19412336 PMC2668767

[54] McAnally AA, Yampolsky LY. Widespread transcriptional autosomal dosage compensation in Drosophila correlates with gene expression level. Genome Biol Evol. 2009;2:44–52. doi: 10.1093/gbe/evp054 20333221 PMC2839349

[55] Lee H, Cho D-Y, Whitworth C, Eisman R, Phelps M, Roote J, et al. Effects of Gene Dose, Chromatin, and Network Topology on Expression in Drosophila melanogaster. PLoS Genet. 2016;12:e1006295. doi: 10.1371/journal.pgen.1006295 27599372 PMC5012587

[56] Zhang Y, Malone JH, Powell SK, Periwal V, Spana E, Macalpine DM, et al. Expression in aneuploid Drosophila S2 cells. PLoS Biol. 2010;8:e1000320. doi: 10.1371/journal.pbio.1000320 20186269 PMC2826376

[57] Larrimore KE, Barattin-Voynova NS, Reid DW, Ng DTW. Aneuploidy-induced proteotoxic stress can be effectively tolerated without dosage compensation, genetic mutations, or stress responses. BMC Biol. 2020;18:117. doi: 10.1186/s12915-020-00852-x 32900371 PMC7487686

[58] Pavelka N, Rancati G, Zhu J, Bradford WD, Saraf A, Florens L, et al. Aneuploidy confers quantitative proteome changes and phenotypic variation in budding yeast. Nature. 2010;468:321–325. doi: 10.1038/nature09529 20962780 PMC2978756

[59] Geistlinger L, da Silva VH, Cesar ASM, Tizioto PC, Waldron L, Zimmer R, et al. Widespread modulation of gene expression by copy number variation in skeletal muscle. Sci Rep. 2018;8:1399. doi: 10.1038/s41598-018-19782-4 29362391 PMC5780461

[60] Malone JH, Cho D-Y, Mattiuzzo NR, Artieri CG, Jiang L, Dale RK, et al. Mediation of Drosophila autosomal dosage effects and compensation by network interactions. Genome Biol. 2012;13:r28. doi: 10.1186/gb-2012-13-4-r28 22531030 PMC3446302

[61] Kacser H, Burns JA. The molecular basis of dominance. Genetics. 1981;97:639–666. doi: 10.1093/genetics/97.3-4.639 7297851 PMC1214416

[62] Collins RL, Brand H, Karczewski KJ, Zhao X, Alföldi J, Francioli LC, et al. A structural variation reference for medical and population genetics. Nature. 2020;581:444–451. doi: 10.1038/s41586-020-2287-8 PMC7334194 32461652

[63] Dennis MY, Eichler EE. Human adaptation and evolution by segmental duplication. Curr Opin Genet Dev. 2016;41:44–52. doi: 10.1016/j.gde.2016.08.001 27584858 PMC5161654

[64] Ohno S. Evolution by Gene Duplication. Springer, Berlin, Heidelberg; 1970.

[65] Dorer DR, Ezekiel DH, Christensen AC. The Triplo-lethal locus of Drosophila: reexamination of mutants and discovery of a second-site suppressor. Genetics. 1995;141:1037–1042. doi: 10.1093/genetics/141.3.1037 8582610 PMC1206827

[66] Boyle MI, Jespersgaard C, Brøndum-Nielsen K, Bisgaard A-M, Tümer Z. Cornelia de Lange syndrome. Clin Genet. 2015;88:1–12. doi: 10.1111/cge.12499 25209348

[67] Yan J, Zhang F, Brundage E, Scheuerle A, Lanpher B, Erickson RP, et al. Genomic duplication resulting in increased copy number of genes encoding the sister chromatid cohesion complex conveys clinical consequences distinct from Cornelia de Lange. J Med Genet. 2009;46:626–634. doi: 10.1136/jmg.2008.062471 19052029 PMC4302738

[68] Collins RL, Glessner JT, Porcu E, Niestroj L-M, Ulirsch J, Kellaris G, et al. A cross-disorder dosage sensitivity map of the human genome. bioRxiv. medRxiv; 2021. doi: 10.1101/2021.01.07.21249419 35917817 PMC9742861

[69] Crow JF. Minor viability mutants in Drosophila. Genetics. 1979;92:s165–72. 488699

[70] Deutschbauer AM, Jaramillo DF, Proctor M, Kumm J, Hillenmeyer ME, Davis RW, et al. Mechanisms of haploinsufficiency revealed by genome-wide profiling in yeast. Genetics. 2005;169:1915–1925. doi: 10.1534/genetics.104.036871 15716499 PMC1449596

[71] Delneri D, Hoyle DC, Gkargkas K, Cross EJM, Rash B, Zeef L, et al. Identification and characterization of high-flux-control genes of yeast through competition analyses in continuous cultures. Nat Genet. 2008;40:113–117. doi: 10.1038/ng.2007.49 18157128

[72] Ohnuki S, Ohya Y. High-dimensional single-cell phenotyping reveals extensive haploinsufficiency. PLoS Biol. 2018;16:e2005130. doi: 10.1371/journal.pbio.2005130 29768403 PMC5955526

[73] Cook DL, Gerber AN, Tapscott SJ. Modeling stochastic gene expression: implications for haploinsufficiency. Proc Natl Acad Sci U S A. 1998;95:15641–15646. doi: 10.1073/pnas.95.26.15641 9861023 PMC28097

[74] Lee H, Cho D-Y, Wojtowicz D, Harbison ST, Russell S, Oliver B, et al. Dosage-Dependent Expression Variation Suppressed on the Drosophila Male X Chromosome. G3. 2018;8:587–598. doi: 10.1534/g3.117.300400 29242386 PMC5919722

[75] Veitia RA. Exploring the etiology of haploinsufficiency. Bioessays. 2002;24:175–184. doi: 10.1002/bies.10023 11835282

[76] Hodgkin J. Fluxes, doses and poisons: molecular perspectives on dominance. Trends Genet. 1993;9:1–2. doi: 10.1016/0168-9525(93)90050-R 8434410

[77] Papp B, Pál C, Hurst LD. Dosage sensitivity and the evolution of gene families in yeast. Nature. 2003;424:194–197. doi: 10.1038/nature01771 12853957

[78] Lambertsson A. The minute genes in Drosophila and their molecular functions. Adv Genet. 1998;38:69–134. doi: 10.1016/s0065-2660(08)60142-x 9677706

[79] Abruzzi KC, Smith A, Chen W, Solomon F. Protection from free beta-tubulin by the beta-tubulin binding protein Rbl2p. Mol Cell Biol. 2002;22:138–147. doi: 10.1128/MCB.22.1.138-147.2002 11739729 PMC134216

[80] Morrill SA, Amon A. Why haploinsufficiency persists. Proc Natl Acad Sci U S A. 2019;116:11866–11871. doi: 10.1073/pnas.1900437116 31142641 PMC6575174

[81] Matharu N, Rattanasopha S, Tamura S, Maliskova L, Wang Y, Bernard A, et al. CRISPR-mediated activation of a promoter or enhancer rescues obesity caused by haploinsufficiency. Science. 2019;363. doi: 10.1126/science.aau0629 30545847 PMC6570489

[82] Chen Z-X, Golovnina K, Sultana H, Kumar S, Oliver B. Transcriptional effects of gene dose reduction. Biol Sex Differ. 2014;5:5. doi: 10.1186/2042-6410-5-5 24581086 PMC3974007

[83] Chen R, Shi L, Hakenberg J, Naughton B, Sklar P, Zhang J, et al. Analysis of 589,306 genomes identifies individuals resilient to severe Mendelian childhood diseases. Nat Biotechnol. 2016;34:531–538. doi: 10.1038/nbt.3514 27065010

[84] Kawauchi S, Calof AL, Santos R, Lopez-Burks ME, Young CM, Hoang MP, et al. Multiple organ system defects and transcriptional dysregulation in the Nipbl(+/-) mouse, a model of Cornelia de Lange Syndrome. PLoS Genet. 2009;5:e1000650. doi: 10.1371/journal.pgen.1000650 19763162 PMC2730539

[85] Leclair NK, Brugiolo M, Urbanski L, Lawson SC, Thakar K, Yurieva M, et al. Poison Exon Splicing Regulates a Coordinated Network of SR Protein Expression during Differentiation and Tumorigenesis. Mol Cell. 2020;80:648–665.e9. doi: 10.1016/j.molcel.2020.10.019 33176162 PMC7680420

[86] Teng X, Dayhoff-Brannigan M, Cheng W-C, Gilbert CE, Sing CN, Diny NL, et al. Genome-wide consequences of deleting any single gene. Mol Cell 2013;52:485–494. doi: 10.1016/j.molcel.2013.09.026 24211263 PMC3975072

[87] El-Brolosy MA, Kontarakis Z, Rossi A, Kuenne C, Günther S, Fukuda N, et al. Genetic compensation triggered by mutant mRNA degradation. Nature. 2019;568:193–197. doi: 10.1038/s41586-019-1064-z 30944477 PMC6707827

[88] El-Brolosy MA, Stainier DYR. Genetic compensation: A phenomenon in search of mechanisms. PLoS Genet. 2017;13:e1006780. doi: 10.1371/journal.pgen.1006780 28704371 PMC5509088

[89] Ma Z, Zhu P, Shi H, Guo L, Zhang Q, Chen Y, et al. PTC-bearing mRNA elicits a genetic compensation response via Upf3a and COMPASS components. Nature. 2019;568:259–263. doi: 10.1038/s41586-019-1057-y 30944473

[90] Serobyan V, Kontarakis Z, El-Brolosy MA, Welker JM, Tolstenkov O, Saadeldein AM, et al. Transcriptional adaptation in Caenorhabditis elegans. Elife. 2020;9. doi: 10.7554/eLife.50014 31951195 PMC6968918

[91] Dickinson ME, Flenniken AM, Ji X, Teboul L, Wong MD, White JK, et al. High-throughput discovery of novel developmental phenotypes. Nature. 2016;537:508–514. doi: 10.1038/nature19356 27626380 PMC5295821

[92] Buhler M, Verdel A, Moazed D. Tethering RITS to a nascent transcript initiates RNAi- and heterochromatin-dependent gene silencing. Cell. 2006;125:873–886. doi: 10.1016/j.cell.2006.04.025 16751098

[93] Keller C, Buhler M. Chromatin-associated ncRNA activities. Chromosome Res. 2013;21:627–641. doi: 10.1007/s10577-013-9390-8 24249576 PMC3855497

[94] Vaschetto LM. miRNA activation is an endogenous gene expression pathway. RNA Biol. 2018;15:826–828. doi: 10.1080/15476286.2018.1451722 29537927 PMC6152443

[95] Mégarbané A, Ravel A, Mircher C, Sturtz F, Grattau Y, Rethoré M-O, et al. The 50th anniversary of the discovery of trisomy 21: the past, present, and future of research and treatment of Down syndrome. Genet Med. 2009;11:611–616. doi: 10.1097/GIM.0b013e3181b2e34c 19636252

[96] Peroos S, Forsythe E, Pugh JH, Arthur-Farraj P, Hodes D. Longevity and Patau syndrome: what determines survival? BMJ Case Rep. 2012;2012. doi: 10.1136/bcr-06-2011-4381 23220825 PMC4543265

[97] Donovan JH, Krigbaum G, Bruns DA. Medical interventions and survival by gender of children with trisomy 18. Am J Med Genet C Semin Med Genet. 2016;172:272–278. doi: 10.1002/ajmg.c.31522 27530709

[98] Davies W. The contribution of Xp22.31 gene dosage to Turner and Klinefelter syndromes and sex-biased phenotypes. Eur J Med Genet. 2021;64:104169. doi: 10.1016/j.ejmg.2021.104169 33610733

[99] Starostik MR, Sosina OA, McCoy RC. Single-cell analysis of human embryos reveals diverse patterns of aneuploidy and mosaicism. Genome Res. 2020;30:814–825. doi: 10.1101/gr.262774.120 32641298 PMC7370883

[100] Shahbazi MN, Wang T, Tao X, Weatherbee BAT, Sun L, Zhan Y, et al. Developmental potential of aneuploid human embryos cultured beyond implantation. Nat Commun. 2020;11:3987. doi: 10.1038/s41467-020-17764-7 32778678 PMC7418029

[101] Bolton H, Graham SJL, Van der Aa N, Kumar P, Theunis K, Fernandez Gallardo E, et al. Mouse model of chromosome mosaicism reveals lineage-specific depletion of aneuploid cells and normal developmental potential. Nat Commun. 2016;7: 11165. doi: 10.1038/ncomms11165 27021558 PMC4820631

[102] McKusick VA. Mendelian Inheritance in Man and its online version, OMIM. Am J Hum Genet. 2007;80:588–604. doi: 10.1086/514346 17357067 PMC1852721

[103] Martin HC, Jones WD, McIntyre R, Sanchez-Andrade G, Sanderson M, Stephenson JD, et al. Quantifying the contribution of recessive coding variation to developmental disorders. Science. 2018;362:1161–1164. doi: 10.1126/science.aar6731 30409806 PMC6726470

[104] Wright CF, Fitzgerald TW, Jones WD, Clayton S, McRae JF, van Kogelenberg M, et al. Genetic diagnosis of developmental disorders in the DDD study: a scalable analysis of genome-wide research data. Lancet 2015;385:1305–1314. doi: 10.1016/S0140-6736(14)61705-0 25529582 PMC4392068

[105] Lancaster MA, Renner M, Martin C-A, Wenzel D, Bicknell LS, Hurles ME, et al. Cerebral organoids model human brain development and microcephaly. Nature. 2013;501: 373–379. doi: 10.1038/nature12517 23995685 PMC3817409

[106] Sharp AJ, Cheng Z, Eichler EE. Structural variation of the human genome. Annu Rev Genomics Hum Genet. 2006;7:407–442. doi: 10.1146/annurev.genom.7.080505.115618 16780417

[107] Hua Y, Krainer AR. Chapter 18—Antisense-Oligonucleotide Modulation of SMN2 Pre-mRNA Splicing. In: Sumner CJ, Paushkin S, Ko C-P, editors. Spinal Muscular Atrophy. Academic Press; 2017. pp. 301–311.

[108] Bachtrog D, Mahajan S, Bracewell R. Massive gene amplification on a recently formed Drosophila Y chromosome. Nat Ecol Evol. 2019;3:1587–1597. doi: 10.1038/s41559-019-1009-9 31666742 PMC7217032

[109] Haig D. Self-imposed silence: parental antagonism and the evolution of X-chromosome inactivation. Evolution. 2006;60:440–447. 16637489

[110] Larsson AJM, Coucoravas C, Sandberg R, Reinius B. X-chromosome upregulation is driven by increased burst frequency. Nat Struct Mol Biol 2019;26:963–969. doi: 10.1038/s41594-019-0306-y 31582851

[111] Deng X, Hiatt JB, Nguyen DK, Ercan S, Sturgill D, Hillier LW, et al. Evidence for compensatory upregulation of expressed X-linked genes in mammals, Caenorhabditis elegans and Drosophila melanogaster. Nat Genet. 2011;43:1179–1185. doi: 10.1038/ng.948 22019781 PMC3576853

[112] Xiong Y, Chen X, Chen Z, Wang X, Shi S, Wang X, et al. RNA sequencing shows no dosage compensation of the active X-chromosome. Nat Genet. 2010;42:1043–1047. doi: 10.1038/ng.711 21102464

[113] Wang Z-Y, Leushkin E, Liechti A, Ovchinnikova S, Mößinger K, Brüning T, et al. Transcriptome and translatome co-evolution in mammals. Nature. 2020;588:642–647. doi: 10.1038/s41586-020-2899-z 33177713 PMC7116861

[114] Chen Y, Chen S, Li K, Zhang Y, Huang X, Li T, et al. Overdosage of Balanced Protein Complexes Reduces Proliferation Rate in Aneuploid Cells. Cell Syst. 2019;9:129–142.e5. doi: 10.1016/j.cels.2019.06.007 31351919

[115] Brennan CM, Vaites LP, Wells JN, Santaguida S, Paulo JA, Storchova Z, et al. Protein aggregation mediates stoichiometry of protein complexes in aneuploid cells. Genes Dev. 2019;33:1031–1047. doi: 10.1101/gad.327494.119 31196865 PMC6672052

[116] Lallemand T, Leduc M, Landès C, Rizzon C, Lerat E. An Overview of Duplicated Gene Detection Methods: Why the Duplication Mechanism Has to Be Accounted for in Their Choice. Genes. 2020;11. doi: 10.3390/genes11091046 32899740 PMC7565063

[117] Kellis M, Birren BW, Lander ES. Proof and evolutionary analysis of ancient genome duplication in the yeast Saccharomyces cerevisiae. Nature. 2004;428: 617–624. doi: 10.1038/nature02424 15004568

[118] Dennis MY, Nuttle X, Sudmant PH, Antonacci F, Graves TA, Nefedov M, et al. Evolution of human-specific neural SRGAP2 genes by incomplete segmental duplication. Cell. 2012;149: 912–922. doi: 10.1016/j.cell.2012.03.033 22559943 PMC3365555

[119] De La Torre AR, Lin Y-C, Van de Peer Y, Ingvarsson PK. Genome-wide analysis reveals diverged patterns of codon bias, gene expression, and rates of sequence evolution in picea gene families. Genome Biol Evol. 2015;7:1002–1015. doi: 10.1093/gbe/evv044 25747252 PMC4419791

[120] Hanada K, Zou C, Lehti-Shiu MD, Shinozaki K, Shiu S-H. Importance of lineage-specific expansion of plant tandem duplicates in the adaptive response to environmental stimuli. Plant Physiol. 2008;148:993–1003. doi: 10.1104/pp.108.122457 18715958 PMC2556807

[121] Ramsey J. Polyploidy and ecological adaptation in wild yarrow. Proc Natl Acad Sci U S A. 2011;108:7096–7101. doi: 10.1073/pnas.1016631108 21402904 PMC3084070

[122] Greenberg MVC, Bourc’his D. The diverse roles of DNA methylation in mammalian development and disease. Nat Rev Mol Cell Biol 2019;20:590–607. doi: 10.1038/s41580-019-0159-6 31399642

[123] Kent LN, Ohboshi S, Soares MJ. Akt1 and insulin-like growth factor 2 (Igf2) regulate placentation and fetal/postnatal development. Int J Dev Biol. 2012;56:255–261. doi: 10.1387/ijdb.113407lk 22562201 PMC3894249

[124] Magklara A, Yen A, Colquitt BM, Clowney EJ, Allen W, Markenscoff-Papadimitriou E, et al. An epigenetic signature for monoallelic olfactory receptor expression. Cell. 2011;145:555–570. doi: 10.1016/j.cell.2011.03.040 21529909 PMC3094500

[125] Zwemer LM, Zak A, Thompson BR, Kirby A, Daly MJ, Chess A, et al. Autosomal monoallelic expression in the mouse. Genome Biol. 2012;13:R10. doi: 10.1186/gb-2012-13-2-r10 22348269 PMC3334567

[126] Deng Q, Ramsköld D, Reinius B, Sandberg R. Single-cell RNA-seq reveals dynamic, random monoallelic gene expression in mammalian cells. Science 2014;343:193–196. doi: 10.1126/science.1245316 24408435

[127] Reinius B, Sandberg R. Random monoallelic expression of autosomal genes: stochastic transcription and allele-level regulation. Nat Rev Genet. 2015;16:653–664. doi: 10.1038/nrg3888 26442639

[128] Vigneau S, Vinogradova S, Savova V, Gimelbrant A. High prevalence of clonal monoallelic expression. Nat Genet. 2018;1198–1199. doi: 10.1038/s41588-018-0188-7 30082785

[129] Marion-Poll L, Forêt B, Zielinski D, Massip F, Attia M, Carter AC, et al. Locus specific epigenetic modalities of random allelic expression imbalance. Nat Commun. 2021;12:5330. doi: 10.1038/s41467-021-25630-3 PMC8429725 34504093

[130] Kumar S, Stecher G, Suleski M, Hedges SB. TimeTree: A Resource for Timelines, Timetrees, and Divergence Times. Mol Biol Evol. 2017;34:1812–1819. doi: 10.1093/molbev/msx116 28387841

[131] Yu G, Smith DK, Zhu H, Guan Y, Lam TT-Y. Ggtree: An r package for visualization and annotation of phylogenetic trees with their covariates and other associated data. Methods Ecol Evol. 2017;8:28–36.

