# Supplementary material for: The good, the bad, and the ugly: Evolutionary and pathological aspects of gene dosage alterations
Source: PLoS Genet. 2021 Dec 9;17(12):e1009906. doi: 10.1371/journal.pgen.1009906 (PMC8659298; doi:10.1371/journal.pgen.1009906)
Supplement: S1 File — (PDF) [file pgen.1009906.s001.pdf]

Dosage Compensation in various species

related to Figure 2, also see Supporting Information of [1]

**Species [Reference]**	**System**	**Dosage Compensation (Footnotes)**
Homo sapiens [2,3]	XY	X inactivation, translation upregulation?
Mus musculus [2,3]	XY	X inactivation, translation upregulation?
Monodelphis domestica [2]	XY	complete
Ornithorhynchus anatinus [2]	XY	none
Gallus gallus [4]	ZW	none[1]
Corvus corone [5]	ZW	none
Ficedula albicollis [6]	ZW	none
Poecilia parae [7]	XY	complete
Gasterosteus aculeatus [8]	XY	none[2]
Cynoglossus semilaevis [9]	ZW	incomplete, 1.3x chromosome-wide?
Anolis carolinensis [10]	XY	complete
Basiliscus vittatus [11]	XY	incomplete[3]
Varanus komodoensis [12]	ZW	none
Apalone spinifera [13]	ZW	sometimes complete[4]
Sistrurus miliarius [14]	ZW	none
Artemia franciscana [15]	ZW	complete
Drosophila melanogaster [16]	XY	complete[5]
Anopheles gambiae [17]	XY	complete
Themira minor [16]	XY	complete[6]
Glossina morsitans [16]	XY	complete
Teleopsis dalmanni [18]	XY	incomplete
Xenos vesparum [19]	XY	partial
Tribolium castaneum [20]	XY	complete[7]
Acyrthosiphon pisum [21]	X0	complete
Papilio xuthus/machaon [22]	ZW	complete
Bombyx mori [22]	ZW	complete
Plodia interpunctella [22]	ZW	complete
Manduca sexta [23]	ZW	complete
Schistosoma mansoni [24]	ZW	complete / none[8]

---

[1] Compensation is achieved by maintaining Y-linked copies of dosage-sensitive genes
[2] Compensation is achieved by maintaining Y-linked copies of dosage-sensitive genes
[3] *n*=10 genes only analyzed, most exhibit compensation, but do not equalize to females levels
[4] Environmentally and developmentally plastic, sometimes tissue-specific, various modes
[5] Many studies, MSL complex-mediated Histone H4 K16 acetylation responsible for upregulation.
[6] Mild upregulation in females and downregulation in males compared to the proto-X levels.
[7] some studies indicate upregulation in XY males and from both XX in females
[8] complete in certain developmental stages, no compensation in others.

Caenorhabditis elegans [25]	X0	complete[9]
Silene latifolia [26,27]	XY	complete / incomplete[10]
Silene pseudotites [28]	XY	none
Silene otites [28]	ZW	partial
Rumex rothschildianus [29]	XY	incomplete
Coccinia grandis [30]	XY	complete[11]
Cannabis sativa [31]	XY	complete[11]
Pristionchus pacificus [32]	X0	complete
Oncorhynchus mykiss [33]	XY	incomplete
Lucilia cuprina [34]	XY	complete
Halyomorpha halys [35]	XY	complete
Homalodisca vitripennis [35]	XY	complete
Oncopeltus fasciatus [35]	XY	complete
Bos taurus [36]	XY	complete
Taeniopygia guttata [37]	ZW	incomplete
Charadrius alexandrinus [38]	ZW	incomplete
Sylvia communis [39]	ZW	incomplete
Heliconius melpomene [40]	ZW	partial
Cydia pomonella [41]	ZW	dosage balance[12]

[9] downregulation of each hermaphrodite X chromosome by 50%
[10] different conclusions in the two studies
[11] more extensive analyses needed
[12] ZZ dampening in males to match Z levels of females, but incomplete.

References

1. Palmer DH, Rogers TF, Dean R, Wright AE. How to identify sex chromosomes and their turnover. Mol Ecol. 2019;28: 4709–4724.

2. Julien P, Brawand D, Soumillon M, Necsulea A, Liechti A, Schütz F, et al. Mechanisms and evolutionary patterns of mammalian and avian dosage compensation. PLoS Biol. 2012;10: e1001328.

3. Wang Z-Y, Leushkin E, Liechti A, Ovchinnikova S, Mößinger K, Brüning T, et al. Transcriptome and translatome co-evolution in mammals. Nature. 2020;588: 642–647.

4. Zimmer F, Harrison PW, Dessimoz C, Mank JE. Compensation of Dosage-Sensitive Genes on the Chicken Z Chromosome. Genome Biol Evol. 2016;8: 1233–1242.

5. Wolf JB, Bryk J. General lack of global dosage compensation in ZZ/ZW systems? Broadening the perspective with RNA-seq. BMC Genomics. 2011;12: 91.

6. Uebbing S, Künstner A, Mäkinen H, Ellegren H. Transcriptome sequencing reveals the character of incomplete dosage compensation across multiple tissues in flycatchers. Genome Biol Evol. 2013;5: 1555–1566.

7. Metzger DCH, Sandkam BA, Darolti I, Mank JE. Rapid Evolution of Complete Dosage Compensation in Poecilia. Genome Biol Evol. 2021;13. doi:10.1093/gbe/evab155

8. White MA, Kitano J, Peichel CL. Purifying Selection Maintains Dosage-Sensitive Genes during Degeneration of the Threespine Stickleback Y Chromosome. Mol Biol Evol. 2015;32: 1981–1995.

9. Chen S, Zhang G, Shao C, Huang Q, Liu G, Zhang P, et al. Whole-genome sequence of a flatfish provides insights into ZW sex chromosome evolution and adaptation to a benthic lifestyle. Nat Genet. 2014;46: 253–260.

10. Marin R, Cortez D, Lamanna F, Pradeepa MM, Leushkin E, Julien P, et al. Convergent origination of a Drosophila-like dosage compensation mechanism in a reptile lineage. Genome Res. 2017;27: 1974–1987.

11. Acosta A, Suárez-Varón G, Rodríguez-Miranda LA, Lira-Noriega A, Aguilar-Gómez D, Gutiérrez-Mariscal M, et al. Corytophanids Replaced the Pleurodont XY System with a New Pair of XY Chromosomes. Genome Biol Evol. 2019;11: 2666–2677.

12. Rovatsos M, Rehák I, Velenský P, Kratochvíl L. Shared Ancient Sex Chromosomes in Varanids, Beaded Lizards, and Alligator Lizards. Mol Biol Evol. 2019;36: 1113–1120.

13. Bista B, Wu Z, Literman R, Valenzuela N. Thermosensitive sex chromosome dosage compensation in ZZ/ZW softshell turtles, Apalone spinifera. Philos Trans R Soc Lond B Biol Sci. 2021;376: 20200101.

14. Vicoso B, Emerson JJ, Zektser Y, Mahajan S, Bachtrog D. Comparative sex chromosome genomics in snakes: differentiation, evolutionary strata, and lack of global dosage compensation. PLoS Biol. 2013;11: e1001643.

15. Huylmans AK, Toups MA, Macon A, Gammerdinger WJ, Vicoso B. Sex-Biased Gene Expression and Dosage Compensation on the Artemia franciscana Z-Chromosome. Genome Biol Evol. 2019;11: 1033–1044.

16. Vicoso B, Bachtrog D. Numerous transitions of sex chromosomes in Diptera. PLoS Biol.

2015;13: e1002078.

17. Rose G, Krzywinska E, Kim J, Revuelta L, Ferretti L, Krzywinski J. Dosage Compensation in the African Malaria Mosquito Anopheles gambiae. Genome Biol Evol. 2016;8: 411–425.

18. Baker RH, Narechania A, DeSalle R, Johns PM, Reinhardt JA, Wilkinson GS. Spermatogenesis Drives Rapid Gene Creation and Masculinization of the X Chromosome in Stalk-Eyed Flies (Diopsidae). Genome Biol Evol. 2016;8: 896–914.

19. Mahajan S, Bachtrog D. Partial dosage compensation in Strepsiptera, a sister group of beetles. Genome Biol Evol. 2015;7: 591–600.

20. Whittle CA, Kulkarni A, Extavour CG. Absence of a Faster-X Effect in Beetles (Tribolium, Coleoptera). G3 . 2020;10: 1125–1136.

21. Richard G, Legeai F, Prunier-Leterme N, Bretaudeau A, Tagu D, Jaquiéry J, et al. Dosage compensation and sex-specific epigenetic landscape of the X chromosome in the pea aphid. Epigenetics Chromatin. 2017;10: 30.

22. Huylmans AK, Macon A, Vicoso B. Global Dosage Compensation Is Ubiquitous in Lepidoptera, but Counteracted by the Masculinization of the Z Chromosome. Mol Biol Evol. 2017;34: 2637–2649.

23. Smith G, Chen Y-R, Blissard GW, Briscoe AD. Complete dosage compensation and sex-biased gene expression in the moth Manduca sexta. Genome Biol Evol. 2014;6: 526–537.

24. Picard MAL, Vicoso B, Roquis D, Bulla I, Augusto RC, Arancibia N, et al. Dosage Compensation throughout the Schistosoma mansoni Lifecycle: Specific Chromatin Landscape of the Z Chromosome. Genome Biol Evol. 2019;11: 1909–1922.

25. Strome S, Kelly WG, Ercan S, Lieb JD. Regulation of the X chromosomes in Caenorhabditis elegans. Cold Spring Harb Perspect Biol. 2014;6. doi:10.1101/cshperspect.a018366

26. Muyle A, Zemp N, Deschamps C, Mousset S, Widmer A, Marais GAB. Rapid de novo evolution of X chromosome dosage compensation in Silene latifolia, a plant with young sex chromosomes. PLoS Biol. 2012;10: e1001308.

27. Papadopulos AST, Chester M, Ridout K, Filatov DA. Rapid Y degeneration and dosage compensation in plant sex chromosomes. Proc Natl Acad Sci U S A. 2015;112: 13021–13026.

28. Martin H, Carpentier F, Gallina S, Godé C, Schmitt E, Muyle A, et al. Evolution of Young Sex Chromosomes in Two Dioecious Sister Plant Species with Distinct Sex Determination Systems. Genome Biol Evol. 2019;11: 350–361.

29. Crowson D, Barrett SCH, Wright SI. Purifying and Positive Selection Influence Patterns of Gene Loss and Gene Expression in the Evolution of a Plant Sex Chromosome System. Mol Biol Evol. 2017;34: 1140–1154.

30. Fruchard C, Badouin H, Latrasse D, Devani RS, Muyle A, Rhoné B, et al. Evidence for Dosage Compensation in Coccinia grandis, a Plant with a Highly Heteromorphic XY System. Genes . 2020;11. doi:10.3390/genes11070787

31. Prentout D, Razumova O, Rhoné B, Badouin H, Henri H, Feng C, et al. An efficient

RNA-seq-based segregation analysis identifies the sex chromosomes of Cannabis sativa. Genome Res. 2020;30: 164–172.

32. Albritton SE, Kranz A-L, Rao P, Kramer M, Dieterich C, Ercan S. Sex-biased gene expression and evolution of the x chromosome in nematodes. Genetics. 2014;197: 865–883.

33. Hale MC, McKinney GJ, Thrower FP, Nichols KM. Evidence of sex-bias in gene expression in the brain transcriptome of two populations of rainbow trout (Oncorhynchus mykiss) with divergent life histories. PLoS One. 2018;13: e0193009.

34. Davis RJ, Belikoff EJ, Scholl EH, Li F, Scott MJ. no blokes Is Essential for Male Viability and X Chromosome Gene Expression in the Australian Sheep Blowfly. Curr Biol. 2018;28: 1987–1992.e3.

35. Pal A, Vicoso B. The X Chromosome of Hemipteran Insects: Conservation, Dosage Compensation and Sex-Biased Expression. Genome Biol Evol. 2015;7: 3259–3268.

36. Ka S, Ahn H, Seo M, Kim H, Kim JN, Lee H-J. Status of dosage compensation of X chromosome in bovine genome. Genetica. 2016;144: 435–444.

37. Itoh Y, Melamed E, Yang X, Kampf K, Wang S, Yehya N, et al. Dosage compensation is less effective in birds than in mammals. J Biol. 2007;6: 2.

38. Moghadam HK, Harrison PW, Zachar G, Székely T, Mank JE. The plover neurotranscriptome assembly: transcriptomic analysis in an ecological model species without a reference genome. Mol Ecol Resour. 2013;13: 696–705.

39. Sigeman H, Ponnikas S, Videvall E, Zhang H, Chauhan P, Naurin S, et al. Insights into Avian Incomplete Dosage Compensation: Sex-Biased Gene Expression Coevolves with Sex Chromosome Degeneration in the Common Whitethroat. Genes . 2018;9. doi:10.3390/genes9080373

40. Walters JR, Hardcastle TJ, Jiggins CD. Sex Chromosome Dosage Compensation in Heliconius Butterflies: Global yet Still Incomplete? Genome Biol Evol. 2015;7: 2545–2559.

41. Gu L, Walters JR, Knipple DC. Conserved Patterns of Sex Chromosome Dosage Compensation in the Lepidoptera (WZ/ZZ): Insights from a Moth Neo-Z Chromosome. Genome Biol Evol. 2017;9: 802–816.
